# Hepatitis Screening and Treatment Campaign in Malaysia-Validation of Low-cost Point of Care Screening Tests and Nucleic Acid Tests for Hepatitis B and C

**DOI:** 10.5005/jp-journals-10018-1273

**Published:** 2019-02-01

**Authors:** Muhammad Radzi AH, Soek S Tan, Rosmawati Mohamed, Fauziah Jaya, Senamjit K, Azlida C Aun, Ghazali A Kutty, Hin S Wong, Rafidah Abdullah, Mohd R Seman, Mamun Al Mahtab, Zaki Morad, Teck-Onn Lim

**Affiliations:** 1 Hospital Sultanah Bahiyah, Alor Setar, Malaysia; 2 Hospital Selayang, Selangor, Malaysia; 3 University Malaya Medical Centre, Kuala Lumpur, Malaysia; 4 Gastroenterology and Hepatology, Hospital Raja Permaisuri Bainun, Ipoh Perak, Malaysia; 5 Hospital, Raja Permaisuri Bainun Ipoh, Perak, Malaysia; 6 Gastroenterology, Hospital Tengku, Ampuan Afzan, Kuantan, Pahang, Malaysia; 7 Nephrologist, Hospital Kuala Lumpur, Malaysia; 8 Hospital Selayang, Selangor, Malaysia; 9 Hospital Temerloh, Pahang, Malaysia; 10 Hospital Tengku, Ampuan Afzan, Kuantan, Pahang, Malaysia; 11 Department of Hepatology, Bangabandhu Sheikh Mujib Medical University, Dhaka, Bangladesh; 12 National Kidney Foundation, Kuala Lumpur, Malaysia; 13 Hepatitis Free Pahang Society, Pahang Malaysia

**Keywords:** Access to treatment, Diagnostic test, Health services, Hepatitis B, Hepatitis B virus dioxy-ribo nucleic acid, Hepatitis C, Hepatitis C virus ribonucleic acid, Nucleic Acid Tests, Point of Care tests, Screening, Validation.

## Abstract

**Background:**

Two major challenges in implementing budget-constrained Hepatitis screening and treatment campaign in Malaysia are the availability of low-cost point of care tests (POCT) and nucleic acid tests (NAT) for hepatitis C virus ribonucleic acid (HCV RNA) and hepatitis B virus dioxyribo nucleic acid (HBV DNA). We evaluated the performance of these tests in this study.

**Methods:**

We conducted a cross-sectional study to evaluate the diagnostic performance of four POCT brands at 12 sites in Malaysia. We assessed the sensitivity and specificity of the POCTs for the detection of HBsAg and anti-HCV in a finger-stick capillary or venepuncture whole-blood samples compared with test results from lab-based enzyme immunoassay (EIA) or chemi-luminescence immunoassay (CLIA) assay as the reference standard. We also conducted a cross-sectional study on 30 to 139 serum specimen panel to evaluate the diagnostic performance of a low-cost in-house Applied Biosystem^®^TaqMan real-time polymerase chain reaction (PCR) assay (ABS) for the detection of HCV RNA and HBV DNA, compare with Roche Cobas^®^ Ampliprep/TaqMan assay (COBAS).

**Results:**

Between March and December 2017, we enroll 295 participants for the evaluation of POCT for HBsAg and another 307 participants for POCT anti-HCV evaluation. Three of the four POCT brands dropped out of evaluation early on account of sub-optimal sensitivity. The sensitivity of the remaining POCT for HBsAg was 95.2%and specificity 100%, while the POCT for anti-HCV has a sensitivity of 98.1% and specificity 100%.

Hepatitis B virus dioxyribo nucleic acid and HCV RNA concentrations detected by the ABS were systematically higher than those measured by COBAS (mean bias +0.10 and +0.17 log10 IU/mL respectively). The 95% limits of agreement between the two assays are -1.28 to 1.47 log10 IU/mL for HBV DNA and –0.41 to 0.75 log10 IU/mL for HCV RNA.

**Conclusion:**

We found adequate evidence for the diagnostic validity of a low-cost POCT for anti-HCV and HBsAg, as well as for an in-house nucleic acid tests (NAT), to provide support for their broader use in our Hepatitis screening and treatment campaign.

**Abbreviations:**

ABS: Applied Biosystem^®^TaqMan real-time PCR assay, CI: Confidence interval, CLD: Chronic liver disease, CLIA: Chemi-luminescence immunoassay, COBAS: Roche Cobas^®^ Ampliprep/ TaqMan assay, DAA: Direct Acting Anti-Viral drugs, EIA: Enzyme immunoassay, HBV: Hepatitis B virus, HCV: Hepatitis C virus, HFPM: Hepatitis Free Pahang Malaysia, LOA: Limits of agreement, LOD: Limit of detection, MOH: Ministry of Health, Malaysia, NAT: Nucleic Acid Tests, POCT: Point of Care Tests, SD: Standard deviation, WHO: World Health Organization

**How to cite this article:** Radzi AHM, Tan SS, Mohamed R, Jaya F, Senamjit K, Aun AC, Kutty GA, Wong HS, Abdullah R, Seman MR, Mahtab MA, Morad Z, Lim TO. Hepatitis Screening and Treatment Campaign in Malaysia-Validation of Low-cost Point of Care Screening Tests and Nucleic Acid Tests for Hepatitis B and C. Euroasian J Hepatogastroenterol, 2018;8(2):101-107.

## INTRODUCTION

According to the Global Burden of Disease Study 2010, chronic liver disease (CLD) ranks ninth among the top 10 major disease burden in Malaysia.^1^ Each year in Malaysia, about 1500 people died of CLD.^2^ Globocan^3^ estimated another 1750 people died of liver cancer, the fourth most common cause of cancer deaths in Malaysia after lung, breast, and colorectal cancers. In South-East Asia, it is estimated that 27% and 23% of deaths from CLD are attributable to Chronic Hepatitis B (HBV) and Hepatitis C virus (HCV) infection respectively, while alcohol accounts for another 19% of CLD deaths.^2^ The estimated prevalent count of HBV carrier in Malaysia is 250,000 adults (prevalence rate 1.5%; children are relatively unaffected because of vaccination which was introduced since 1989), and another 400,000 people have been infected by HCV (prevalence rate 2%). Hence Chronic HBC and HCV are likely major causes of CLD and liver cancer in Malaysia, though admittedly in the absence of population-based epidemiologic studies, these estimates are derived from studies on blood donors, patients on dialysis or from single tertiary institutions^4-17^ or were modeled estimates.^18-20^

Recent therapeutic advances have rendered both chronic HBV and HCV treatable. HCV, in particular, is curable with modern direct acting anti-viral (DAA) therapies.^21^ In spite of being an upper middle-income country, screening services for HBV and HCV in Malaysia remain under-developed, and access to modern antiviral therapies is even more limited.^22^ There is a need for concerted public actions to address hepatitis as a significant public health concern. However, there is little progress on the public policy front to allocate significant healthcare resources to support hepatitis screening and treatment services in Malaysia. In response, a nongovernmental organization, the Hepatitis Free Pahang Malaysia (HFPM),^23^ was recently established to mobilize local community organizations, health care providersand individual concerned citizens to address Hepatitis health; specifically to raise awareness about HBV and HCV, to provide low cost public screening services and improve access to costly treatments for chronic HCV and HBV in Malaysia. The HFPM has launched its hepatitis campaign ona pilot scale in Pahang since August 2017 and more recently in other parts of Malaysia. The campaign is entirely funded by charity and by HFPM partners comprising local social or faith-based organizations and local healthcare providers as part of their community outreach efforts.

For a low budget charity funded campaign to effectively and ethicallyconduct public screeningand treatment for HBV and HCV on a large scale, we must overcome five major challenges:

Effective social marketing to raise public awareness about HBV and HCV through a variety of channels and media, and to engage the public and inviting them to come forward for screening at our health fairs or opportunistically at participating primary care and pharmacy outlets. This is currently conducted by HFPM together with our partners. To date in our pilot campaign, we have screened 1636 individuals, and 27 people were confirmed to be positive (19 HBsAg+ and eight anti-HCV +).

Public screening entails testing huge number of people in the community. It must necessarily employ robust low-cost POCT which is simple to perform (minimal training and no equipment required) and which provides a result on the spot.^24^ These tests are available but costly. Based on our budgetary consideration, the cost of POCT to sustain our campaign should be below USD 0.50 per person screened.

Subjects who are screened positive from the above-mentioned public campaign will require confirmatory lab-based serological tests as well as nucleic acid tests (NAT) for HCV RNA and HBV DNAtoguide treatment decision. These tests are available but very costly. Based on our budgetary consideration, the cost of NAT should be below USD 20 per test.

Subjects who are confirmed positive with detectable HCV RNA or high HBV DNA levels, among other considerations, will need anti-viral treatment. These medicines are very costly. We follow Australian lead^25^ in using low cost personally imported generic medicines^26^ from India and Bangladesh. However, given the uncertain safety and efficacy of these imported medicines, we have established a patient registry to track the outcomes of treated patients including to determine the sustained virological response (SVR) rates achieved. The registry also serves as an active surveillance system to monitor the safety of these drugs and to collect sample drugs from the diverse supply sources to measure their composition.

In scaling up the Hepatitis campaign, a large number of patients with chronic HBV and HCV will be identified, and they will require medical care. In Malaysia, these patients have hitherto been managed by specialist Gastroenterologists, the supply of whom is minimal. For example, in Pahang with a population of 1.6 million and where the pilot campaign is ongoing, there is only one Gastroenterologist in the public hospital and 2 in private, all are located far away in the capital city Kuantan. In line with current WHO guidelines,^27,28^ we mobilize the local primary care workforce where the campaign is conducted to deliver the medical care for the patients with uncomplicated chronic HBV or HCV who are identified through our campaign.

### In this Study, we aimed to Evaluate

The diagnostic performance of POCTs donated by or purchased from a variety of manufacturers on both finger-stick capillary as well as venepuncture whole-blood samples. The performance results will guide the selection of low-cost POCT for subsequent large-scale use in our public screening campaign.

The diagnostic performance of low-cost HCV RNA and HBV DNA tests for broader use in the treatment campaign.

## METHODS

### Validation of POCT for Detecting Anti-HCV and HBsAg

We conducted a cross-sectional study to evaluate the diagnostic performance of POC tests acquired from 4 manufacturers between March and December 2017. The Ministry of Health’s (MOH) Medical and Research Ethics Committee approved the study, and all patients gave written informed consent.

### Study Patients

Participants for this study were enrolled from 12 sites in Malaysia. These sites comprise five Medical/Gastroenter-ology outpatient clinics and 7 hemodialyses (HD) centers.

To estimate the test sensitivity, we enroll patients aged 18 or older who were positive for anti-HCV or HBsAg. Patients’ HBV or HCV status were verified by the presence of positive results from lab tests using enzyme immunoassay (EIA) and besid es for anti-HCV, chemilu-minescence immunoassay (CLIA). Exclusion criteria were a history of HBV vaccination or undetectable HCV RNA.

To estimate the test specificity, we enrolledsubjects from the public who had come forward to attend HBV and HCV screening and who were tested negative for anti-HCV or HBsAgusing enzyme immunoassay (EIA).

### POCT Tests under Evaluation and Procedures

We identified four manufacturers who agreed to supply POCTs at a low cost to meet our budgetary constraint. POCTs from these sources use the immune-chromato-graphic method in a lateral flow device to detect anti-HCV antibodies or HBsAg in whole blood collected by finger stick, or serum, plasma or whole-blood collected by vene-puncture. In these devices, human plasma-derived anti-hepatitis B surface antibodies or synthetic recombinant HCV antigens (Core, NS3, NS4, NS5) are immobilized on a single test line on a nitrocellulose membrane. HBsAg or anti-HCV antibodies in the samples reactive with these antibodies or antigens respectively are visualized by colloidal goldlabeled protein.

We purchased, or manufacturers donated POCTs for this validation study. The four manufacturers are All Test Biotech Hangzhou China, Hangzhou Voyage Medical Nantong China, Encode Medical Zhuhai China and Labratorium Hepatica Mataram, Indonesia. After initial evaluation on 20 to 50 patients, it had become evident that POCTs from only one manufacturer (All Test Biotech) was likely to meet our minimum test sensitivity requirement of 90%. We continued the evaluation only for POCTs from this manufacturer.

Either finger-stick capillary or venepuncture whole-blood samples were collected from all subjects enrolled. The POCT procedures were performed according to the manufacturers’ instructions. The reading times was 20 minutes, and tests should not be interpreted after 20 minutes. A POCT was interpreted as negative if a control line was present (regardless of intensity) with no corresponding test line. The appearance of a control line and a test line indicated a positive result. An unclear or missing control line indicated an invalid result, regardless of test line presence. In the event of the invalid result, the POCT was repeated until a valid result was obtained.Medical staff from each participating site performed the POCT. All medical staff had received prior training on the test procedure and interpretation. Only a single staff performed the test and read the result. Interoperator variability in test performance or interpretation of test results was not considered in this evalua tion.

### Statistical Methods

We assessed the sensitivity and specificity of POCT for the detection of HBsAg and anti-HCV in a finger-stick capillary or venepuncture whole-blood samplescom-pared with test result from lab-based EIA or CLIA assay as the reference standard. Given the prevalence of chronic HBV or HCV among enrolled subjects is 50%, for sensitivity and specificity of 99%, and margin of error of 2.0%, a minimum sample size of 190 would provide a 95% confidence intervals (CI) of 95 to 100 0% for the estimates of sensitivity and 95 to 100 0% for specificity.

We estimate the 95% CIs for the sensitivity and specificity based on normal or Poisson approximations to the binomial distribution, as appropriate.^29^ Continuous variables were expressed as means with SD or medians with interquartile ranges (IQR).

### Lab Validation of HCV RNA and HBV DNA tests

We also conducted a cross-sectional study to evaluate the diagnostic performance of a low cost in-house NAT for the detection of HCV RNA and HBV DNA between June and December 2017. The Ministry of Health’s (MOH) Medical and Research Ethics Committee approved the study.

### Study Samples

The study is conducted on serum specimen panel which was originally collected from patients with chronic HBV or HCV who had undergone testing for HCV RNA and HBV DNA as part of their routine medical care. We opportunistically use their leftover specimens for this validation study. The HBV DNA panel has 30 serum specimens from 2 laboratories in Malaysia, while the HCV RNA panel has 139 serum specimensfrom laboratories in Malaysia (30 samples), India (99 samples) and Myanmar (10 samples). The serum specimen was frozen at -70°C until they were thawed for this study.

### NAT Tests under Evaluation and Procedures

Hepatitis C virus ribonucleic acid and HBV DNA were measured in stored serum samples usingin-house Applied Biosystem^®^ TaqMan real-time PCR assay (ABS), the lower limit of detection (LOD) for HCV RNA and HBV DNA are 25 IU/mL and 10 IU/mL respectively. We compare these results with Roche Cobas^®^ Ampliprep/TaqMan assay (COBAS) for HCV RNA and HBV DNA as the reference standard (LOD for HCV RNA and HBV DNA are 15 IU/mL and 20 IU/mL respectively).

### Statistical Methods

We used Bland-Altman difference plot^30^ to assess the limits of agreement(LOA) in the quantification of serum HBV DNA and HCV RNA between the in-house ABS assay compared with COBAS assay as the reference.

Assuming the mean difference between the two assays is zero, and SD 0.25 log10 IU/mL, a minimum sample size of 30 specimens would provide a 95% CI of +0.15 log10 IU/mL. All results are reported in log10 units. Data for specimens having results below the lower limit of detection (LOD) were imputed using the midpoint between zero and LOD.

**Table 1:** **Table 1:** Characteristics of patients enrolled in the validation study of anti-HCV and HBsAg POCT for use in public screening

		*Patients positive for HBsAg*		*Patients positive for anti-HCV*		*Persons negative* *for HBsAg* *and anti-HCV*	
		N=145		N=157		N=150	
Mean Age (SD), year		50 (13)		52 (12)		52(12)	
Gender							
Male, %		68%		66%		40%	
Female, %		32%		34%		60%	
Ethnicity							
Malay, %		44%		57%		10%	
Chinese, %		49%		32%		83%	
Indian, %		2%		11%		6%	
Others, %		4%		0%		3%	
Sites							
Medical/Gastroenterology clinic, %		74%		43%			
Hemodialysis centres, %		26%		57%		-	
Individual sites							
Hospital RPB Ipoh		38%		22%		-	
Hospital SHAS Temerloh		24%		20%		-	
Hospital TAA Kuantan		23%		22%		-	
Hospital Kuala Lumpur		11%		24%		-	
Hospital Sungai Buloh & Others		4%		3%		-	
Others		0%		8%		-	

## RESULTS

Table 1 shows the characteristics of the subjects enrolled in the validation study of anti-HCV and HBsAg POCT for use in public screening. Their mean age was 51 years. 6 7% of the patients were male. Most patients (74%) positive for HBsAg were enrolled from Medical and Gastroenterology clinics, while patients positive for anti-HCV were mostly (57%) enrolled from Hemodialysis centers.

Tables 2 and 3 show the diagnostic performance of the POCT for HBsAg and anti-HCV respectively. The POCT for detecting HBsAg has a sensitivity of 95.2% and specificity 100%, giving an overall accuracy of 97.6%. The POCT for detecting anti-HCVhas a sensitivity of 98.1% and specificity 100%, giving an overall accuracy of 99.0%. These results provide adequate evidence of the diagnostic validity of POCT for anti-HCV and HBsAg for use in public screening.

**Figs 1A and B:**
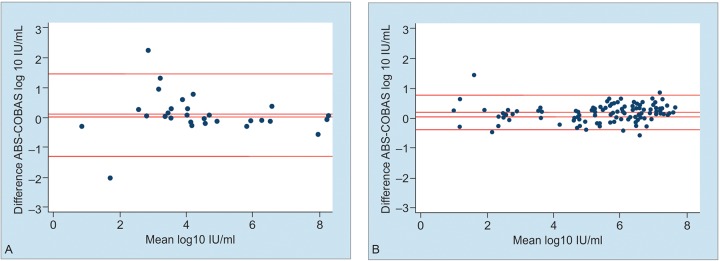
Bland-Altman bias plot of differences. (A) Applied Biosystem TaqMan assay for HBV DNA in serum samples compared with Roche Cobas Ampliprep/TaqMan assay. N = 30, bias 0.10, 95% limits of agreement -1.28 to 1.47; (B) Applied Biosystem TaqMan assay for HCV RNA in serum samples compared with Roche Cobas Ampliprep/TaqMan assay. N = 139, bias 0.17, 95% limits of agreement –0.41 to 0.75

Figure 1 shows the Bland-Altman plot. HBV DNA concentrations detected by the ABS assay were a mean of 0.10 (SD 0.69) log10 IU/mL higher than those measured by the COBAS assay. The limits of agreement indicate that 95% of the differences between the two assays are between -1.23 and 1.47 log10 IU/mL.

**Table 2:** **Table 2:** Diagnostic performance of POCT for detecting HBsAg validated against positive HBsAg by EIA or CLIA as the diagnostic standard

		*Positive HBsAg by EIA*		*Negative* *HBsAg by* *EIA*	
Positive HBsAg by POCT		138 (95.2%)		0 (0%)	
Negative HBsAg by POCT		7 (4.8%)		150 (100%)	
Total		145 (100%)		150 (100%)	

**Table 3:** **Table 3:** Diagnostic performance of POCT for detecting anti-HCV validated against positive Anti-HCV by EIA or CLIA as the diagnostic standard

		*Positive anti-* *HCV by EIA or CLIA*		*Negative anti-HCV by EIA or CLIA*	
Positive Anti-HCV by POCT		154 (98.1%)		0 (0%)	
Negative Anti-HCV by POCT		3 (1.9%)		150 (100%)	
Total		157 (100%)		150 (100%)	

HCV RNA concentrations detected by the ABS assay were a mean of 0.17 (SD 0.29) log10 IU/mL higher than those measured by the COBAS assay. The limits of agreement indicate that 95% of the differences between the two assays are between–0.41 and 0.75log10 IU/mL.

## DISCUSSION

We conducted a diagnostic performance study on four low-cost POC Ts for detecting HBsAg and anti-HCV in whole-blood collected by finger-stick or venepuncture compared with EIA or CLIA assay as the reference standard. Consistent with results from previous studies,^31^ we found a wide range of sensitivity, and evaluation on three of the four selected POCTs were abandoned early on account of suboptimal sensitivity.

Fortunately, we identify one POCT with sensitivity and specificity of 95.2%and 100% for detecting HBsAg. These results are consistent if not better than results from 30 studies on 33 POCTs conducted in 23 countries with varying prevalence. The pooled estimate of the sensitivity was 90.0% (95% CI: 89.1-90.8) and pooled specificity 99.5% (95% CI: 99.4-99.5).^31^ Our sensitivity result seems higher than those reported in published studies to date, which may be because we excluded subjects with a history of HBV vaccination. Of the seven subjects in this study who had a false negative result on the POCT for HBsAg, one had anti-HBs, one had anti-HBe, five had low HBV DNA (<10,000 IU/mL), two had seroconverted to HBeAg^-^.

We also identify one POCT with sensitivity and specificity of 98.1% and 100% respectively for detecting anti-HCV. This is also consistent with previous studies which have found that POCT for anti-HCV performed much better than for HBsAg. A systematic review of 32 studies on 25 POCT brands reported a pooled sensitivity-and specificity of 99% (95% CI 98-100%) and 100% (95% CI100-100%) respectively.^32^

We also conducted a diagnostic performance study on a low-cost in-house Applied Biosystem^®^ TaqMan assay-for the detection of HCV RNA and HBV DNA in stored serum panel compared with Roche Cobas^®^ Ampliprep/ TaqMan assay as the reference. For the quantification of both HBV DNA and HCV RNA, results from our in-house assay were systematically higher (positive bias of 0. 10 and 0.17 log10 IU/mL respectively) than those from the COBAS assay. The range of their 95% limits of agreement 2.75 and 1.16 log10 IU/mL respectively, however, are comparable to previous studies comparing two NAT methods.^33,34^ The limits of agreement indicate that 95% of the differences between Applied Biosystem^®^ TaqMan assay and the Roche Cobas^®^ Ampliprep/TaqMan assay is between -1.28 and 1.47 log10 IU/mL (0.05 to 29.5 IU/ mL) for HBV DNA, and between–0.41 and 0.75 log10 IU/ mL (0.39 to 5.6 IU/mL) for HCV RNA. These differences are not clinically significant.

One limitation of this study is that the participants in this study were enrolled from medical clinics or dialysis centers. They may not be representative of the target population in our public screening campaign. Our results may not be generalizable to the broader population setting, and ongoing monitoring of the tests’ performance during the public screening campaign is warranted.

In conclusion, our results on the POCTs evaluated in this study provide support for broader use in our Hepatitis public screening campaign. Similarly, the results on the diagnostic performance of the NATs give us the confidence to adopt these low-cost NATs for broader use to support our Hepatitis treatment campaign.

## DECLARATIONS

### Ethics Approval and Consent

The Ministry of Health’s (MOH) Medical and Research Ethics Committee approved the study. All patients enrolled for the validation study of POCT for detecting anti-HCV and HBsAg gave written informed consent

## AUTHOR’S CONTRIBUTIONS

MRAH, TSS, RM, FJ, SK, ACA, GAK, WHS, RA, MRS, MAM, ZM contributed to the subject matter expertise. They also contributed to the writing of the manuscript. LTO conceived the idea behind this study and contributed to the study design, data analysis and interpretation, report writing and subject matter expertise. All authors read and approved the final manuscript.
